# Contrasting Magnetic Structures in the Quaternary Sulfides Ba_2_FeMS_5_ (M = Sb, Bi)

**DOI:** 10.1021/acs.inorgchem.4c03770

**Published:** 2024-11-25

**Authors:** Bradley C. Sheath, Stanislav Savvin, Simon J. Clarke

**Affiliations:** † Department of Chemistry, University of Oxford, Inorganic Chemistry Laboratory, South Parks Road, Oxford OX1 3QR, United Kingdom; ‡ Institut Laue-Langevin, 71 Avenue des Martyrs CS 20156, Grenoble 38042, France; § Instituto de Nanociencia y Materiales de Aragón, Facultad de Ciencias, CSIC − Universidad de Zaragoza, C/Pedro Cerbuna 12, Zaragoza 50009, Spain

## Abstract

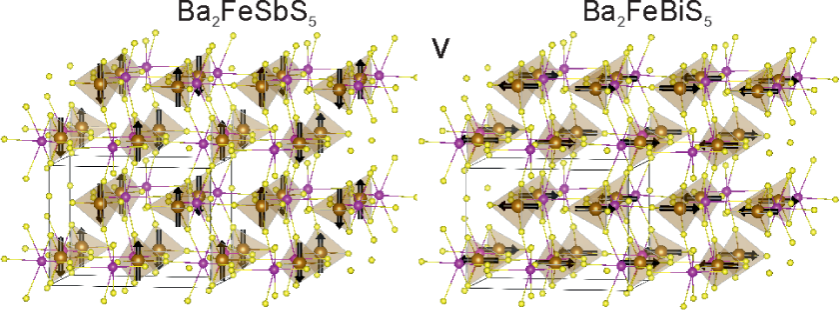

Ba_2_FeSbS_5_ and Ba_2_FeBiS_5_ are two isostructural quaternary sulfides that crystallize in the *Pnma* space group with four formula units per unit cell. Ba_2_FeSbS_5_ has lattice parameters *a* = 12.08609(3) Å, *b* = 8.83426(2) Å, and *c* = 8.89114(2) Å, and Ba_2_FeBiS_5_ has *a* = 12.09610(3) Å, *b* = 8.89281(2) Å, and *c* = 8.82437(2) Å at room temperature. They comprise infinite [FeMS_5_]^4–^ (M = Sb, Bi) chains, where Fe^3+^ is present in FeS_4_ tetrahedra and M^3+^ ions reside in edge-sharing MS_6_ distorted octahedra, each of which shares an edge with an FeS_4_ tetrahedron. Powder neutron diffraction measurements confirm the presence of long-range antiferromagnetic order of the Fe^3+^ moments in both materials, where Fe–S···S–Fe super-superexchange interactions which act along the direction of the [FeMS_5_]^4–^ (M = Sb, Bi) chains are the driving force for this antiferromagnetic order. The magnetic Bragg reflections reside on a *k*-vector with *k* = (1/2 0 1/2), and the relative orientations of the moments are similar in the two cases One significant difference is that the moments are aligned along the crystallographic *b*-axis in Ba_2_FeSbS_5_, whereas in Ba_2_FeBiS_5_ they lie along the longer crystallographic *a*-axis, reflecting the weak directional preference of the Fe^3+^ moments. Furthermore, an additional incommensurately modulated ordering of the Fe^3+^ moments is suggested for Ba_2_FeSbS_5_ (but not Ba_2_FeBiS_5_) by the appearance of small additional magnetic Bragg peaks in the neutron diffraction data, which may be a consequence of greater magnetic frustration in Ba_2_FeSbS_5_.

## Abstract 2

Ba_2_FeSbS_5_ and Ba_2_FeBiS_5_ differ in their magnetic structures likely reflecting the different levels of magnetic frustration determined computationally.

## Introduction

Materials containing sulfide anions can adopt different crystal structures and exhibit different properties to those containing oxide anions. S^2–^ is a less electronegative and more polarizable anion than O^2–^ and the S^2–^ 3p band lies higher in energy than the O^2–^ 2p band. Incorporating sulfide anions rather than oxide anions into a solid can therefore tune many of the characteristics of the compound. Metal-anion bonding is generally more covalent and this can result in the formation of novel structure types–such as the stabilization of layered structures in sulfides. It can also bring about different magnetic exchange pathways, altering the intrinsic magnetism displayed by materials. The electronic band gap between the valence and conduction bands usually decreases in magnitude as the anion is changed from oxide to sulfide, which is why many sulfides are electronic semiconductors rather than insulators. Sulfides have potential uses as photovoltaics,^1^ thermoelectrics^2,3^ and battery cathode materials.^4,5^

Geng et al.^6^ originally reported Ba_2_FeSbS_5_ and Ba_2_FeBiS_5_. Both materials are isostructural, crystallize in the *Pnma* space group and adopt the same structure type as a high-pressure phase of Ba_3_FeS_5_.^7^ UV–vis–NIR optical absorption measurements revealed that these quaternary sulfides are semiconductors with band gaps of 0.95 and 1.28 eV for Ba_2_FeSbS_5_ and Ba_2_FeBiS_5_ respectively.^6^ These were predicted to be charge transfers from the S 3p valence band to the Fe 3d conduction band from first-principle DFT calculations.^6^ Clear antiferromagnetic transitions with Néel temperatures of 13 K (Ba_2_FeSbS_5_) and 35 K (Ba_2_FeBiS_5_) were observed in the magnetometry data,^6^ which encouraged us to explore the long-range magnetic ordering in these systems further using powder neutron diffraction, as Koo and Whangbo^8^ had suggested from a computational investigation that the systems could show different magnetic structures as a consequence of different levels of magnetic frustration. The selenide analogues were reported by J. Wang et al.^9^ and the magnetic structure of the selenide Ba_2_FeSbSe_5_ was reported by Maier et al.^10^ and will also be discussed below. The selenide analogues Ba_2_FeSbSe_5_ and Ba_2_FeBiSe_5_ have been reported^11,12^ to show amorphous-to-crystalline phase changes under irradiation and anomalous changes in thermal conductivity coincident with the antiferromagnetic ordering.

## Experimental Section

### Synthesis

3g samples of Ba_2_FeSbS_5_ and Ba_2_FeBiS_5_ were synthesized from BaS (Aldrich 99.9%), Fe (Alfa Aesar 99.998%), Sb (Alfa Aesar 99.999%)/Bi (Aldrich 99.999%) and S (Alfa Aesar 99.999%) in the ratio of 2:1:1:5. The reagents were thoroughly ground together using an agate pestle and mortar until the mixtures appeared homogeneous. The powders were then pressed into pellets, placed in alumina crucibles and sealed inside evacuated silica tubes. During the tube sealing process, the lower part of the tube–where the crucibles were residing–was submerged in liquid nitrogen to prevent the evaporation of sulfur. The Ba_2_FeSbS_5_ mixture was heated at 400 °C for 24h (0.1 °C/min ramping rate) then 700 °C for 24h (0.5 °C/min ramping rate) and the Ba_2_FeBiS_5_ mixture was heated at 400 °C for 24h (0.1 °C/min ramping rate) then 750 °C for 24h (0.5 °C/min ramping rate). The 400 °C heating step and slow ramping rates were employed to ensure that the sulfur reacted before reaching a high vapor pressure.

### Diffraction

X-ray powder diffraction (XRPD) data for detailed structural analysis were collected on beamline I11^13^ at the Diamond Light Source, Harwell, UK using 30 min scans with 0.824512(5) Å X-rays (calibrated using a Si standard) with the high-resolution Multi-Analyzer Crystal (MAC) detector. Neutron powder diffraction (NPD) was carried out on the D2B^14^ (λ = 1.594 Å) and D1B^15^ (λ = 2.52 Å) instruments at the Institut Laue-Langevin (ILL), Grenoble, France where approximately 2 g of each material was loaded into vanadium cans and data were obtained at various temperatures between 2 and 300 K using a standard ILL “Orange” cryostat to cool down and warm up the samples. D2B was used to collect data at 2 K only, whereas on D1B many data sets were collected while sweeping the temperature continuously on warming. 57 data sets were measured for Ba_2_FeSbS_5_ between 2 and 30 K. 71 data sets were measured for Ba_2_FeBiS_5_ between 2 and 50 K. The XRPD and NPD data were analyzed by Rietveld refinement using the TOPAS Academic V6 software.^16^ Magnetic structures were determined with the aid of the ISOTROPY suite^17^ and structures were visualized in Vesta.^18^

### Magnetometry

Magnetization measurements against temperature and field were carried out using a Quantum Design MPMS-3 SQUID magnetometer. Around 20 mg of material was accurately weighed and placed into a gelatin capsule. This capsule was secured inside a plastic straw and then loaded into the magnetometer. Measurements were performed between 2 and 300 K to investigate the magnetic behavior of the samples. In order to extinguish the effects of any small amount of ferromagnetic impurity when determining the temperature dependence of the magnetic susceptibility, the moment of the sample was measured as a function of temperature at fields of 5 T, and 4 and 3 T, above the saturation of ferromagnetic impurities such as elemental Fe, then the gradient of these three measurements (which was confirmed to be linear at each temperature apart from at the lowest temperatures as described below) was used as the magnetic susceptibility of the main phase and plotted as a function of temperature and used in the Curie–Weiss fitting well above the long-range magnetic ordering transitions. No impurities were detected in the XRPD and NPD data for either compound, however it is not uncommon for miniscule amounts of magnetic impurity to greatly affect magnetization curves. From room temperature magnetization against field measurements (Figure S1), an estimated 1 × 10^–7^ moles and 5 × 10^–8^ moles of Fe per mole of the quaternary sulfide were inferred for Ba_2_FeSbS_5_ and Ba_2_FeBiS_5_ respectively.

## Results and Discussion

### Compositions and Crystal Structures

**Figure 1:**
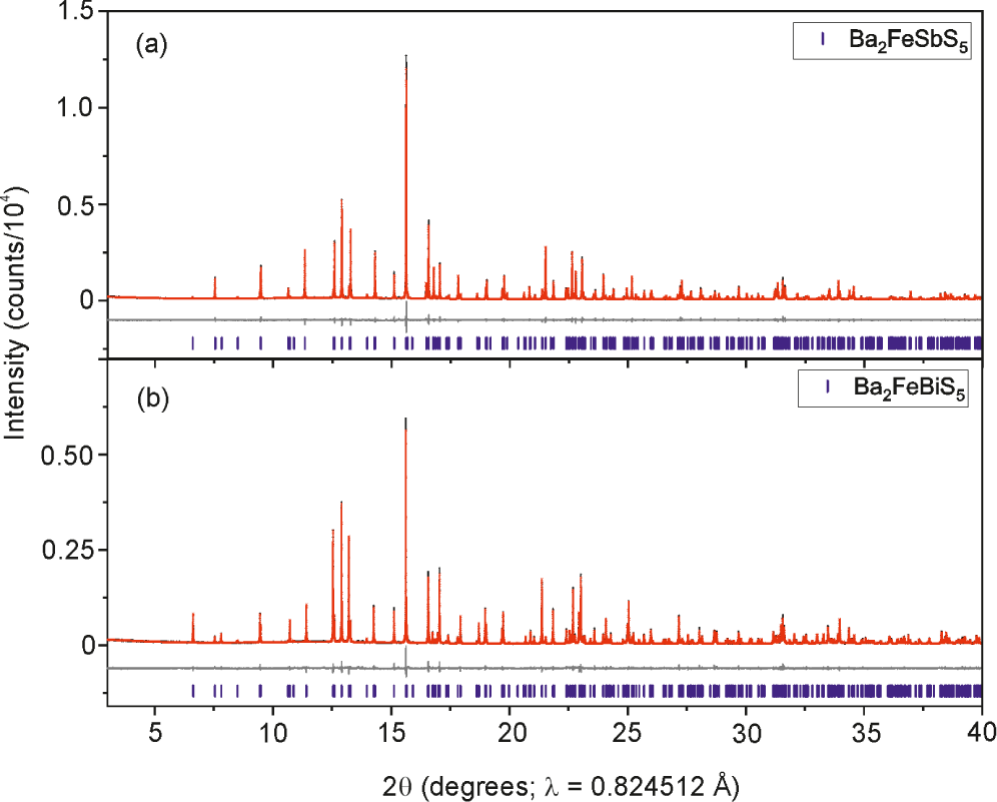
Rietveld refinements against synchrotron XRPD patterns of (a) Ba_2_FeSbS_5_ and (b) Ba_2_FeBiS_5_ measured at 300 K using the MAC detector on I11. Observed (black), calculated (red), and difference (gray) curves are shown. *R*_wp_: 7.733% for Ba_2_FeSbS_5_ and *R*_wp_: 8.401% for Ba_2_FeBiS_5_.

**Figure 2:**
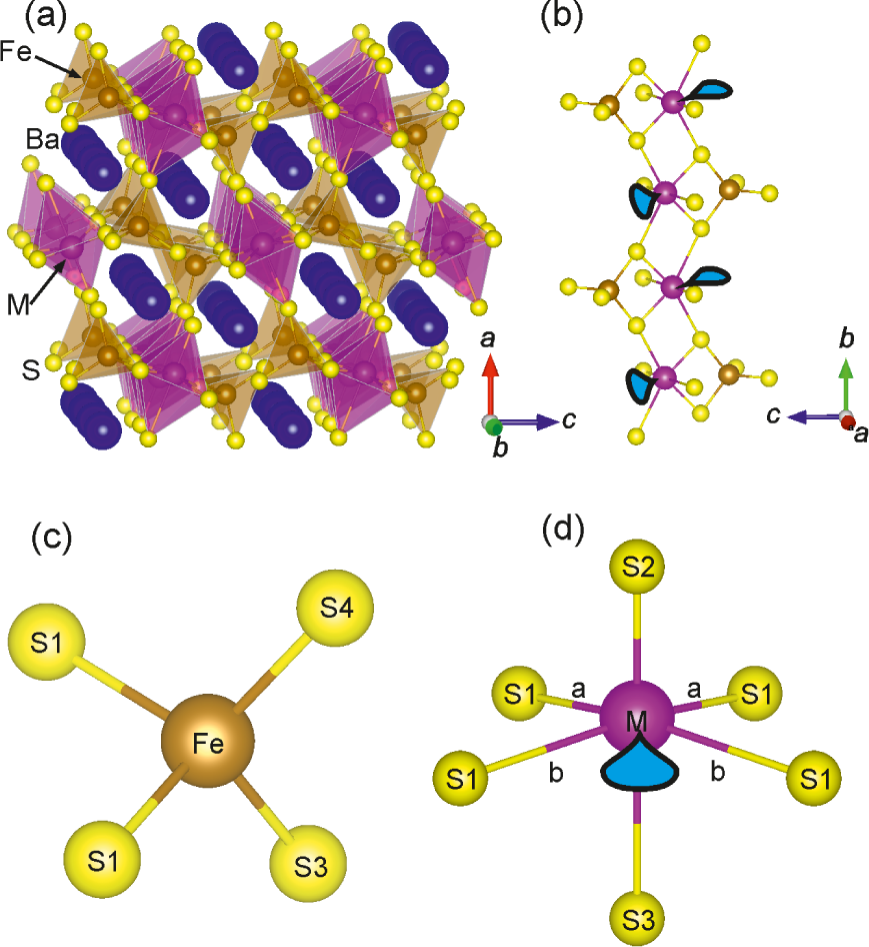
Structure of Ba_2_FeMS_5_ (M = Sb, Bi) (a) with the FeS_4_ (brown) and MS_6_ (purple) polyhedra highlighted and (b) showing only the infinite [FeMS_5_]^4–^ (M = Sb, Bi) 1D chains which run through the structure. (c) The FeS_4_ tetrahedra and (d) the MS_6_ (M = Sb, Bi) distorted octahedra. Bond distances are given in Table 1. The teardrop shapes depict the stereochemically active lone pair.

Ba_2_FeSbS_5_ and Ba_2_FeBiS_5_ were obtained in the form of black powders. Rietveld refinement against room temperature XRPD data (Figure 1) do not indicate the presence of any side phases or stoichiometry away from the target formulas. These materials crystallize in the orthorhombic *Pnma* space group (structure given in Figure 2a) and can be viewed as consisting of infinite [FeMS_5_]^4–^ (M = Sb, Bi) chains (see Figure 2b), where Fe^3+^ is tetrahedrally coordinated by sulfide anions and a distorted octahedral arrangement of sulfide anions surrounds M^3+^. Figure 2c,d depicts these two polyhedra. The edge-sharing MS_6_ polyhedra form the backbone of these chains and the FeS_4_ tetrahedra share one of their edges with one of the edges of an MS_6_ polyhedron. The FeS_4_ polyhedra are arranged on alternating sides of the MS_6_ polyhedral chains, and this allows adjacent Sb/Bi stereoactive lone pairs, most likely positioned between the two M-S1b bonds, to be situated on opposite sides of the backbone.

### Structure Refinement

The lattice parameters, atomic positions and a selection of bond lengths, refined from synchrotron XRPD data (Figure 1), are given in Table 1. A comparison between these values refined from 300 K XRPD data and those refined from 2 K NPD data is given in Tables S1 and S2.

**Table 1:** Refinement Results from Synchrotron XRPD Patterns at 300 K

	Ba_2_FeSbS_5_	Ba_2_FeBiS_5_
Diffractometer	I11 (MAC)	I11 (MAC)
Wavelength (Å)	0.824512	0.824512
Temperature (K)	300	300
Space Group	*Pnma*	*Pnma*
*a* (Å)	12.08609(3)	12.09610(3)
*b* (Å)	8.83426(2)	8.89281(2)
*c* (Å)	8.89114(2)	8.82437(2)
*V* (Å^3^)	949.321(3)	949.224(3)
Fe–S1 [2]^a^ (Å)	2.286(3)	2.289(4)
Fe–S3 [1] (Å)	2.219(5)	2.250(6)
Fe–S4 [1] (Å)	2.193(5)	2.170(6)
M-S1a [2] (Å)	2.499(3)	2.655(4)
M-S1b [2] (Å)	3.136(3)	3.061(4)
M-S2 [1] (Å)	2.424(4)	2.532(5)
M-S3 [1] (Å)	3.241(4)	3.209(5)
Fe BVS^b^	+3.10(4)	+3.08(5)
M BVS	+3.46(5)	+3.41(5)

Atom^c^	Site	*x*	*y*	*z*	*U*_iso_ (Å^2^)
Ba_2_FeSbS_5_					
Ba	8*d*	0.32277(6)	0.00774(9)	0.37767(6)	0.0160(3)
Sb	4*c*	0.02265(9)	0.25	0.5182(1)	0.0134(4)
Fe	4*c*	0.0943(2)	0.25	0.1705(3)	0.0066(8)
S1	8*d*	0.0527(3)	0.0501(3)	0.3236(3)	0.015(1)
S2	4*c*	0.2099(4)	0.25	0.6157(5)	0.012(2)
S3	4*c*	0.2737(4)	0.25	0.1173(5)	0.018(2)
S4	4*c*	0.4936(4)	0.25	0.5347(5)	0.018(1)
Ba_2_FeBiS_5_					
Ba	8*d*	0.32224(7)	0.0099(1)	0.38338(9)	0.0042(4)
Bi	4*c*	0.01744(7)	0.25	0.53310(8)	0.0044(4)
Fe	4*c*	0.0942(3)	0.25	0.1715(3)	0.0000(9)
S1	8*d*	0.0551(3)	0.0457(4)	0.3199(4)	0.003(1)
S2	4*c*	0.2159(4)	0.25	0.6245(6)	0.003(2)
S3	4*c*	0.2760(4)	0.25	0.1177(6)	0.002(2)
S4	4*c*	0.4963(4)	0.25	0.5345(5)	0.005(1)

a The numbers in the square brackets give the multiplicity of each type of bond; coordination environments are shown in Figure 2.

b BVS = bond valence sum.

c labeling of the S atoms is the system adopted by Geng et al.^6^

### Crystal Structures

The unit cell volumes of the two compounds are almost identical due to the fact that the longer *a*-axis is largely unchanged upon going from the Sb to the Bi analogue and the shorter *b*-axis and *c*-axis are also very similar in magnitude (although *b* is ∼0.7% longer than *c* for Ba_2_FeBiS_5_ and vice versa for Ba_2_FeSbS_5_). This can be attributed to the similar ionic radii of Sb^3+^ and Bi^3+^ due to the lanthanide and relativistic contractions and the minimal change in structure between the two samples. The stereoactive ns^2^ lone pair appears to be directed between the M-S1b bonds, in the space where no atoms reside. It is this lone pair that gives the M cation the distorted octahedral coordination environment via a second order Jahn–Teller distortion involving ns/np mixing mediated by the anion valence orbitals.^19^

The Fe Bond Valence Sum (BVS) is very close to +3 for both compounds (Table 1), which is the expected formal oxidation state of Fe in these systems based on the formulas. The fact that these values are comparable reflects the similar Fe–S bond lengths and arrangement of the S^2–^ anions around the Fe centers in the two materials. The M cations have three short M–S distances and three much longer ones (Table 1). With the much longer M–S1b and M–S3 distances included, so that the coordination number of M is six, the calculated BVS values for M are slightly larger than +3. With only the three short distances included, the M BVS values are +3.01(3) (M = Sb) and +2.70(4) (M = Bi).

### Magnetometry

**Figure 3:**
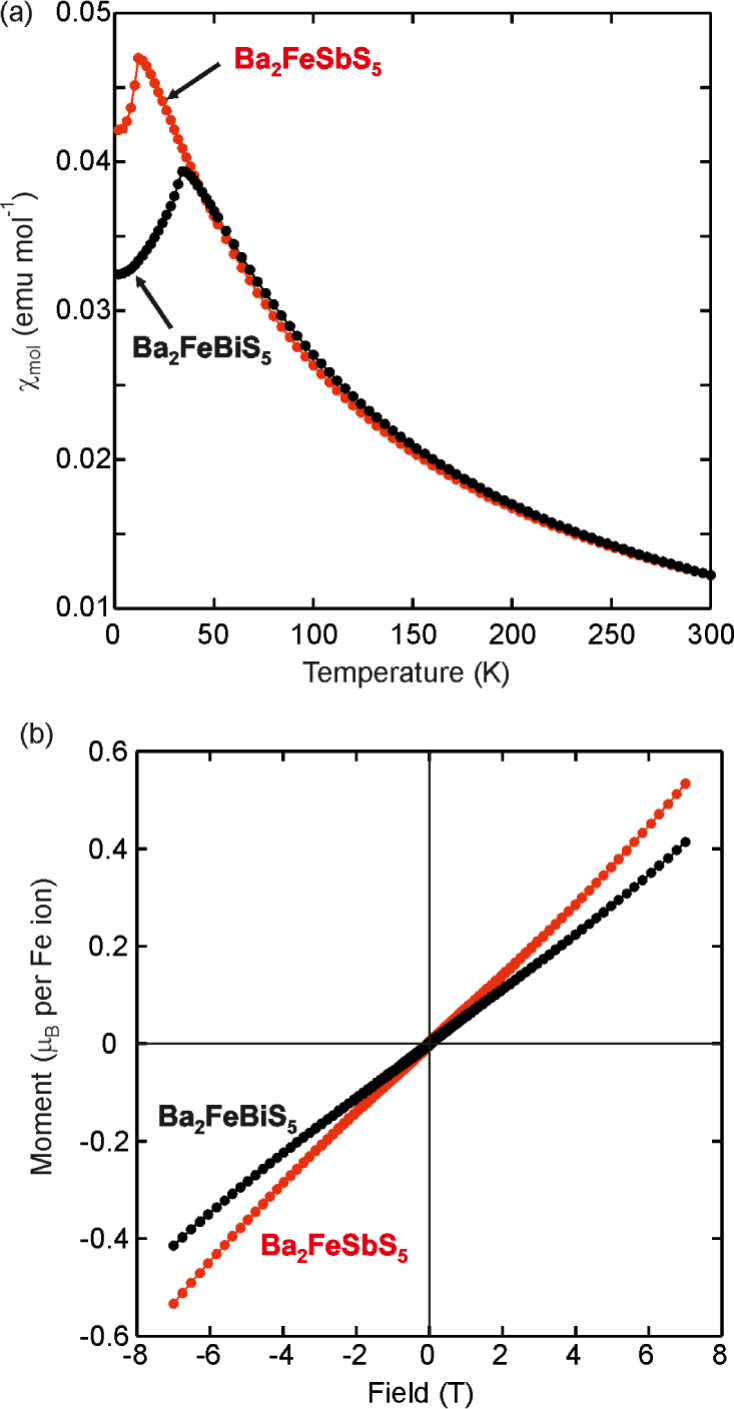
Comparison of (a) the magnetic susceptibilities against temperature (measured according to the protocol described above to eliminate the effect of small ferromagnetic impurities (see Figure S1)) and (b) the magnetization isotherms measured at 5 K for Ba_2_FeBiS_5_ (black) and Ba_2_FeSbS_5_ (red) (a very tiny hysteresis due to the small ferromagnetic impurity present in each case (see Figure S1) is not resolved in this plot).

Magnetometry measurements on the two compounds are compared in Figure 3. As previously reported by Geng et al.,^6^ both show antiferromagnetic transitions – Ba_2_FeSbS_5_ at *T*_*N*_ = 13(1) K and Ba_2_FeBiS_5_ at 35 (1) K (Figure 3a) based on the susceptibility maxima. The 5 K magnetization isotherms in Figure 3b are very similar. Both are symmetric with respect to the origin and at these low temperatures, in the magnetically ordered regimes, they show a slight increase in gradient at higher fields. This increase could plausibly indicate a field-induced moment reorientation of the long-range ordered state at much higher fields, which could be explored further using neutron powder diffraction in high magnetic fields. This behavior in the low-temperature isotherms is reminiscent of that reported in the selenide analogues, Ba_2_FeSbSe_5_ and Ba_2_FeBiSe_5_.^9^

Curie–Weiss fits (see Figures S2 and S3) to the magnetometry data in the range 250–300 K, well above the magnetic transitions, produce values for the effective magnetic moment, μ_eff_, of 5.99(3) μ_B_ and 5.90(1) μ_B_ per Fe^3+^ ion for Ba_2_FeSbS_5_ and Ba_2_FeBiS_5_ respectively. These values are close to the spin-only effective moments predicted for a high-spin *d*^5^ ion (μ_eff_ = 5.92 μ_B_), confirming that Fe^3+^ ions populate the Fe sites. S. Wang et al.^20^ report μ_eff_ = 5.94(3) μ_B_ for Ba_2_FeBiS_5_ single crystals. The values are larger, and much closer to the spin-only value for Fe^3+^ than those reported by Geng et al.^6^ (5.09(1) and 5.66(3) μ_B_ for Ba_2_FeSbS_5_ and Ba_2_FeBiS_5_ respectively). The Weiss temperatures, θ_W_, which are a measure of the average strength of the exchange interactions are determined to be −67(1) K for Ba_2_FeSbS_5_ and −55(1) K for Ba_2_FeBiS_5_, comparable to the values of Geng et al.^6^ and the −60.27(6) K for Ba_2_FeBiS_5_ found by S. Wang et al.,^20^ consistent with antiferromagnetism. As Koo and Whangbo point out,^8^ the much larger ratio of the magnitude of the Weiss temperature to the antiferromagnetic ordering temperature in Ba_2_FeSbS_5_ suggests that it is much more highly frustrated than Ba_2_FeBiS_5_. The selenide analogues originally reported by J. Wang et al.^9^ are reported to have higher magnetic ordering and Weiss temperatures than the sulfides: Ba_2_FeSbSe_2_: *T*_N_ = 58(1) K, θ_W_ ∼ −125 K; Ba_2_FeBiSe_2_: *T*_N_ = 79(2) K, θ_W_ ∼ −135 K, so Ba_2_FeSbS_5_ is the most highly frustrated of the four systems based on the |θ_W_|/*T*_N_ values summarized in Table 2.

**Table 2:** Comparison of Magnetic Properties from Magnetic Susceptibility Measurements

Compound	*T*_N_ (K)	μ_eff_ (μ_B_) per Fe^3+^ ion	Weiss constant (θ_W_) (K)	|θ_W_|/*T*_N_	Ref
Ba_2_FeSbS_5_	13(1)	5.99(3)	–67(1)	5.2	This work
Ba_2_FeBiS_5_	35(1)	5.90(1)^a^	–55(1)^a^	1.6	This work
Ba_2_FeSbSe_2_	58(1)	5.3(1)	–125(1)	2.2	(9)
Ba_2_FeBiSe_2_	79(2)	6.0(1)	–135(1)	1.7	(9)

a S. Wang et al.^20^ report similar values.

### Antiferromagnetic Ordering in Ba_2_FeSbS_5_

**Figure 4:**
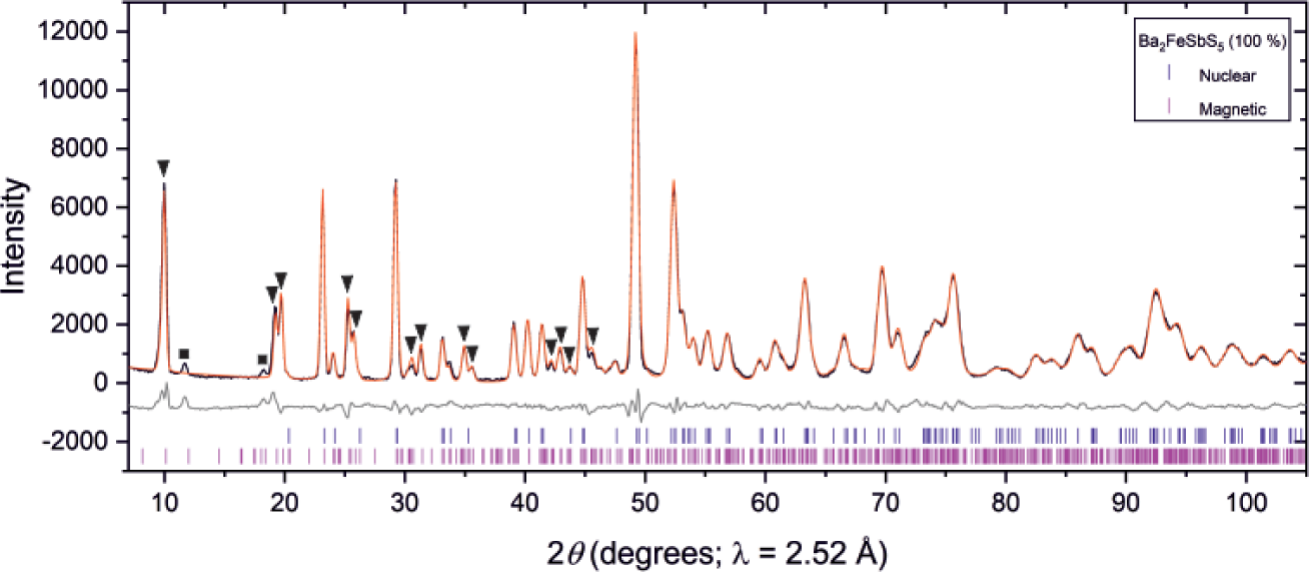
NPD pattern of Ba_2_FeSbS_5_ measured at 1.9 K on the D1B instrument at the ILL showing the observed (black), calculated (red), and difference (gray) curves. The black triangles denote magnetic reflections with *k* = (1/2 0 1/2), and the black squares indicate two low intensity magnetic reflections which cannot be accounted for using a commensurate magnetic ordering model. *R*_wp_: 4.504%.

**Figure 5:**
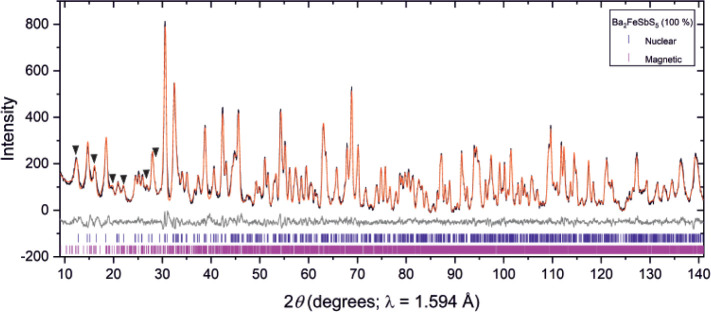
NPD pattern of Ba_2_FeSbS_5_ measured at 2 K on the D2B instrument at the ILL showing the observed (black), calculated (red), and difference (gray) curves. The black triangles denote magnetic reflections with *k* = (1/2 0 1/2). *R*_wp_: 2.650%.

Low temperature NPD measurements on Ba_2_FeSbS_5_ show the presence of reflections at high *d*-spacings which cannot be accounted for by the nuclear model alone. Instead, these peaks can be explained by long-range antiferromagnetic ordering of the Fe moments. The vast majority of these peaks are positioned on a *k*-vector with *k* = (1/2 0 1/2) (the *U* point of the Brillouin Zone) and these are denoted by the black triangles in Figures 4 and 5. Two further additional peaks of lower intensity are present in Figure 4 (labeled by black squares, see also Figure S4). These appear on cooling at the same temperature as the other magnetic reflections, but they cannot be accounted for using magnetic modes represented by commensurate *k*-vectors. It should be pointed out here that these two weak peaks are only visible when collecting diffraction data on the extremely high signal/noise D1B instrument (Figure 4) which focuses on the long *d*-spacing region and that they are not visible in the diffraction pattern collected on D2B using 1.6 Å neutrons (Figure 5). In the D2B data, a large background signal is present up to approximately 2θ = 10°, which prevents the lowest angle Bragg peak from being easily identifiable and hence the refinement in Figure 5 begins at 2θ = 9°.

**Figure 6:**
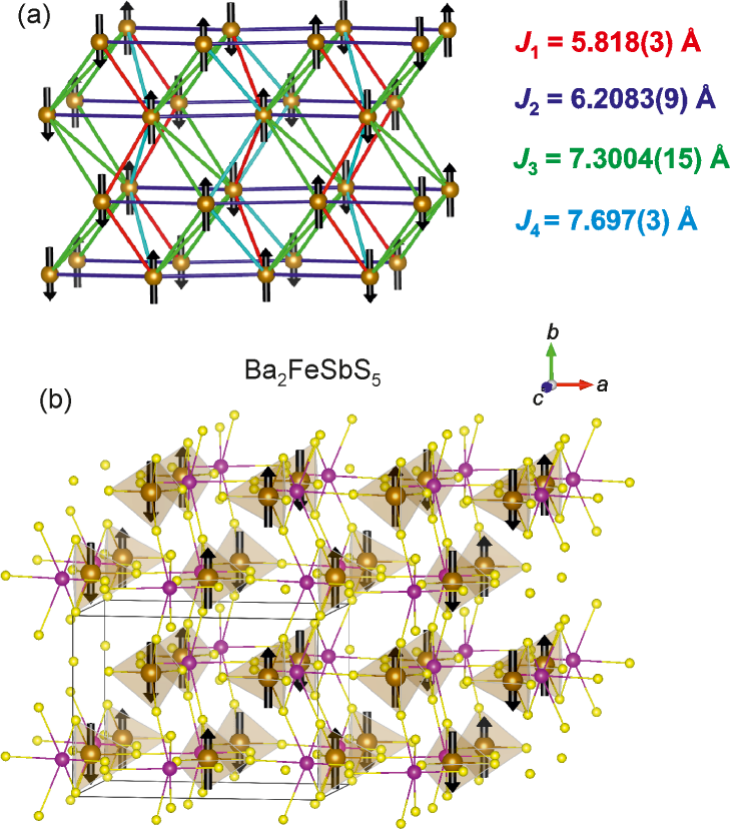
Magnetic model for Ba_2_FeSbS_5_. In (a), distances are given for the magnetic interactions between Fe ions refined from the NPD data collected at 2 K on D2B, and the definition of the different *J* couplings is as given in ref^10^. In (b), the nuclear unit cell is highlighted by the solid black lines, and atoms are Fe: brown; M (M = Sb): purple; S: yellow. Ba atoms are omitted for clarity.

To a good approximation, but neglecting the two small peaks noted in Figure 4, the long-range magnetic ordering in Ba_2_FeSbS_5_ can be represented by the model in Figure 6. This model was determined by testing the activation of different possible magnetic modes on the Fe sites compatible with the *k*-vector (1/2 0 1/2), using the ISOTROPY suite^17^ interfaced with Topas Academic. It was found that the best fit to the magnetic reflections was accounted for by the activation of two mU2^–^U3^–^ (a,b) modes. In the refinement all Fe sites were assumed to have the same moment as they are crystallographically identical. The magnetic model can be described as a collinear arrangement of antiferromagnetically aligned moments, which are directed along the crystallographic *b*-axis, and it is similar to that found for Ba_2_FeSbSe_2_.^10^ It is described (see Table S3) in the magnetic space group^21^*P*_*a*_2_1_/*m* (No. 11.55) in the Belov–Neronova–Smirnova (BNS) scheme,^22^ which corresponds to space group 11.6.64 in the Opechowski–Guccione (OG) scheme.^23^

**Figure 7:**
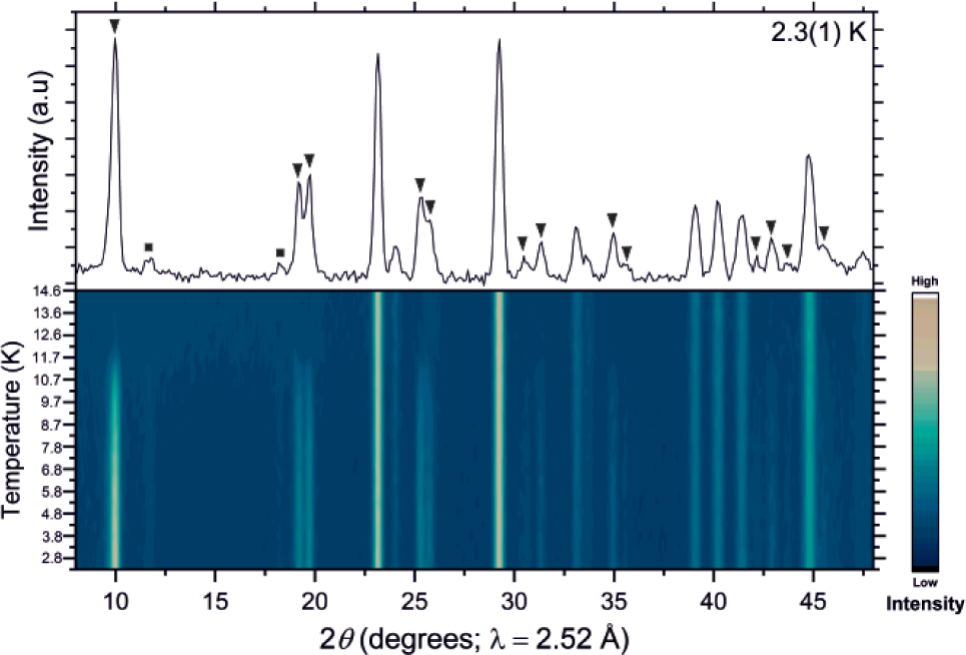
Plot of the NPD data collected for Ba_2_FeSbS_5_ from 2 to 15 K on D1B at the ILL. The black triangles denote magnetic reflections with *k* = (1/2 0 1/2), and the black squares indicate the two low intensity magnetic reflections which cannot be accounted for using a commensurate magnetic ordering model, but which emerge at the same temperature as the other magnetic reflections. See also Figure S4.

All magnetic peaks decrease in intensity until a Néel temperature of 12.1(3) K (consistent with the value from the magnetic susceptibility measurements). Figure S5a shows the evolution of the long-range ordered moment for Ba_2_FeSbS_5_ determined from refinement against all the magnetic Bragg peaks apart from the two small peaks which arise from the presumed incommensurate ordering. The saturated long-range ordered moment at low temperatures is 3.56(2) μ_B_ and this decrease from the expected 5 μ_B_ for a high-spin *d*^5^ localized moment with full magnetic long-range order is likely to be due to covalency in the Fe–S bonds. It can be seen in Figure 7 that all magnetic Bragg peaks appear simultaneously below *T*_N_, including the two low intensity peaks which we assume arise from an incommensurate component of the ordering. There is no obvious shift in the *d*-spacing values of these two low intensity peaks with temperature (beyond that expected for thermal expansion) (see Figure S4). Determination of a critical exponent for the evolution of the ordered moment (Figure S5a) was attempted, however a reliable value was not obtained due to a dearth of points in the region just below *T*_N_.

We attempted to account for the two low-intensity magnetic reflections, labeled by the black squares in Figures 4 and 7, by introducing an incommensurately modulated spin-density wave with wavevector *k* = 0.186 1/2 1/2 superimposed on the commensurate ordering of the Fe spins. An example of a possible magnetic model is depicted in Figure S6. However, the small number of these peaks and their low intensity meant that a unique solution (including the wavevector) for the apparent incommensurate component of the long-range magnetic ordering could not be obtained, and we conclude that a single crystal neutron diffraction measurement would be needed to resolve this.

### Magnetic Ordering in Ba_2_FeBiS_5_

**Figure 8:**
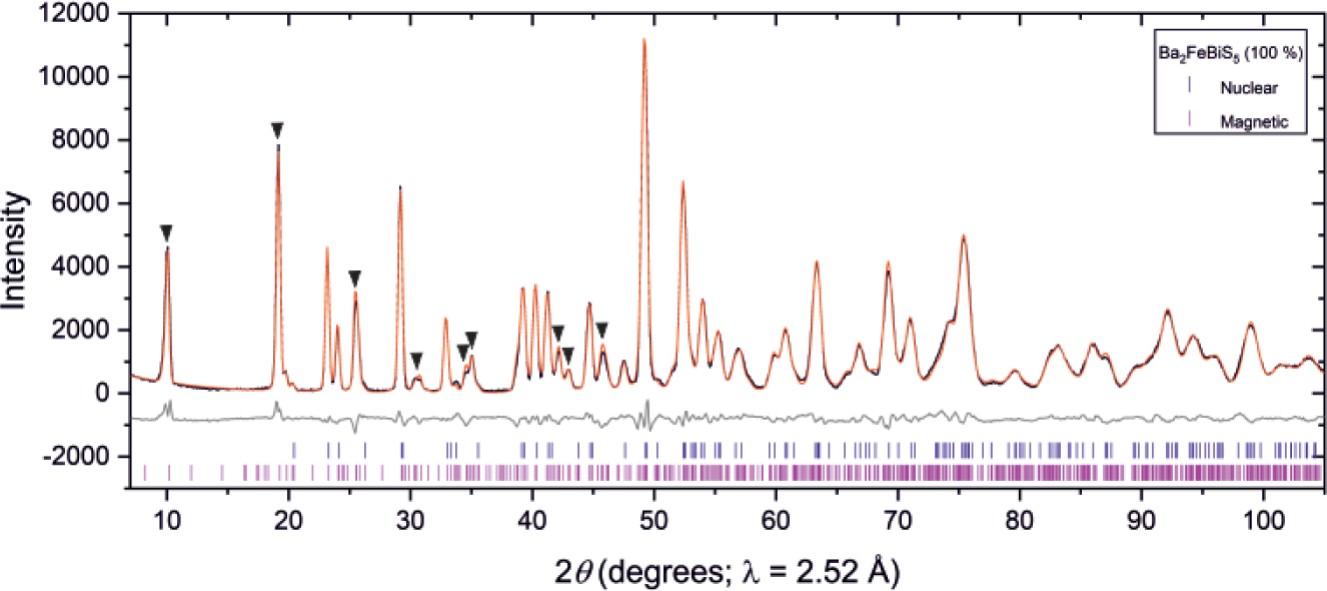
NPD pattern of Ba_2_FeBiS_5_ measured at 1.9 K on D1B at the ILL showing the observed (black), calculated (red), and difference (gray) curves. The black triangles denote magnetic reflections with *k* = (1/2 0 1/2), and no reflections remain unindexed; *R*_wp_: 4.216%.

**Figure 9:**
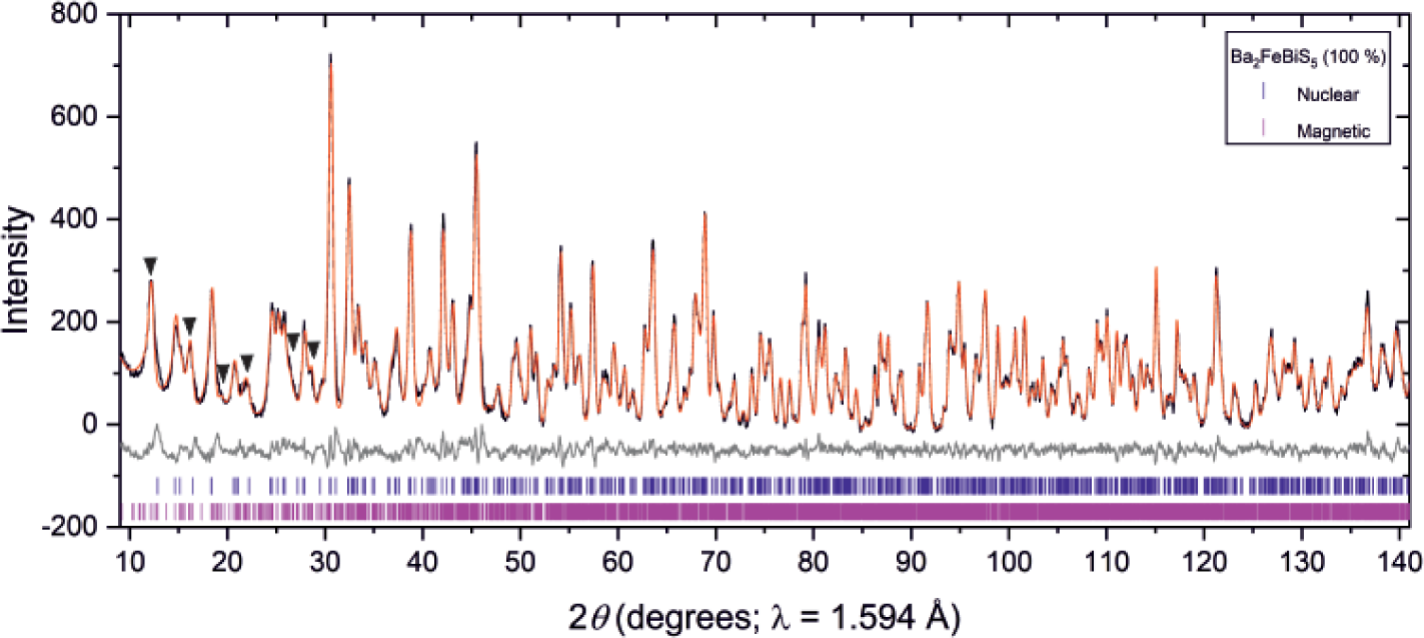
NPD pattern of Ba_2_FeBiS_5_ measured at 2 K on D2B at the ILL showing the observed (black), calculated (red), and difference (gray) curves. The black triangles denote magnetic reflections with *k* = (1/2 0 1/2). *R*_wp_: 2.936%.

**Figure 10:**
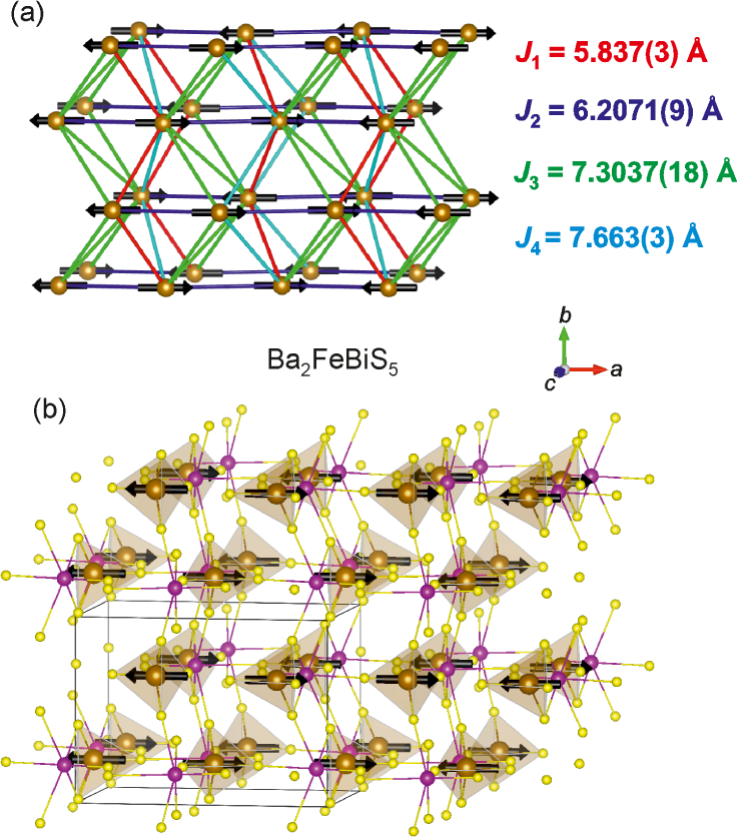
Magnetic model for Ba_2_FeBiS_5_. In (a), Fe–Fe distances are given for the magnetic interactions, refined from the NPD data collected at 2 K on D2B, and the definition of the different *J* couplings is as given in ref^10^. In (b), the nuclear unit cell is highlighted by the solid black lines. Atoms are Fe: brown; M (M = Bi): purple; S: yellow. Ba atoms are omitted for clarity.

Low temperature NPD studies carried out on Ba_2_FeBiS_5_ also reveal reflections at high *d*-spacings due to long-range antiferromagnetic ordering of the Fe moments. These are denoted by the black triangles in Figures 8 and 9 and are once again located on a *k*-vector (1/2 0 1/2). In this case, analysis of the intensities showed that, in contrast to Ba_2_FeBiS_5_, simultaneous refinement of two mU1^–^U4^–^(a,b) modes, again with all the crystallographically equivalent Fe ions carrying moments of equal magnitude, accounted for all the magnetic scattering. The best fitting model for these data is given in Figure 10 and is described in the Shubnikov group *P*_*c*_2_1_/*c* (No. 14.82) in the BNS^22^ scheme (11.7.65 in the OG^23^ scheme) (see Table S3). Like in Ba_2_FeSbS_5_ and Ba_2_FeSbSe_5_^10^ there is a collinear arrangement of the moments which are antiferromagnetically aligned, but in contrast to the two Sb-containing cases, in Ba_2_FeBiS_5_, the moments are directed along the crystallographic *a*-axis–perpendicular to the moments in Ba_2_FeSbS_5_ and Ba_2_FeSbSe_5_.^10^ Unlike in the case of Ba_2_FeSbS_5_ there are no peaks left unindexed by the commensurate magnetic model.

**Figure 11:**
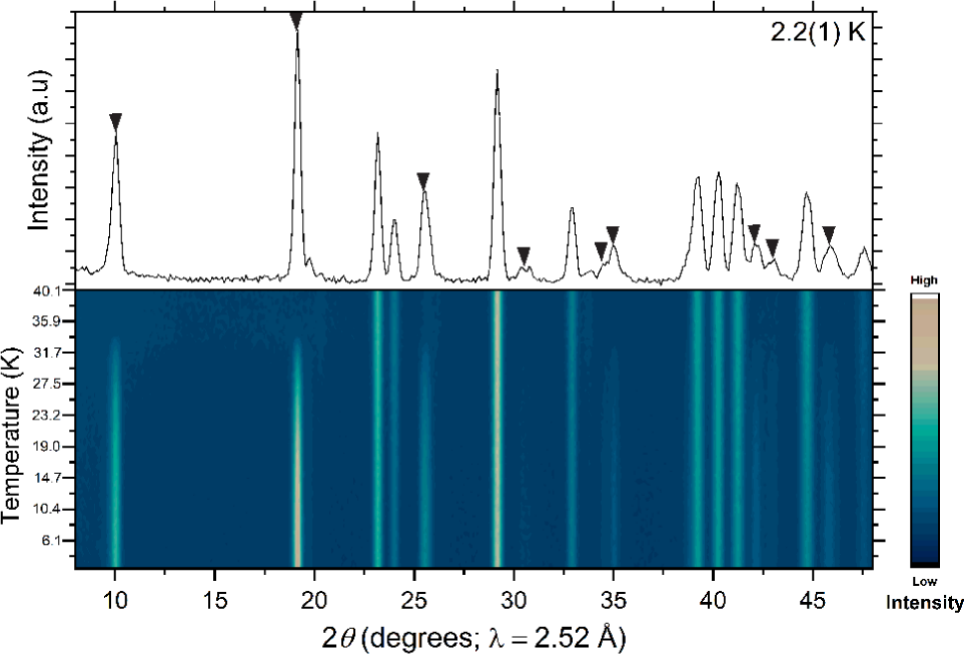
Plot of the NPD data collected for Ba_2_FeBiS_5_ from 2 to 40 K on the D1B instrument at the ILL. The black triangles denote magnetic reflections with *k* = (1/2 0 1/2).

The magnetic peaks for Ba_2_FeBiS_5_ decrease in intensity until a Néel temperature of 35(1) K, consistent with the magnetometry and the saturated long-range ordered moment is 3.78(3) μ_B_, as illustrated by the plot in Figure S5b. As for Ba_2_FeSbS_5_ (ordered moment of 3.6(1) μ_B_), the reduction of this localized magnetic moment from the expected 5 μ_B_ is likely to be due to covalency in the Fe–S bonds. For comparison, the Fe moment in CuFeS_2_, which contains Fe^3+^ ions in FeS_4_ tetrahedra, has been reported as 3.85 μ_B_^24^ and 3.42(7) μ_B_^25^ in different measurements, whereas the Fe moment in KFeO_2_, which contains Fe^3+^ ions in FeO_4_ tetrahedra, is reported to be slightly larger 3.99 μ_B_^26^ and 3.910(18) μ_B_^27^ in different measurements. In Ba_2_FeSbSe_2_ each Fe^3+^ ion carries a reported long-range ordered moment of 4.13(4) μ_B_.^10^ It can be seen in Figure 11 that all magnetic Bragg peaks in Ba_2_FeBiS_5_ appear simultaneously below *T*_N_ = 35(1) K. The attempt to extract a critical exponent for the temperature dependence of the long-range ordered magnetic moment (Figure S5b) did not produce a satisfactory fit.

### Magnetic Ordering Comparison

**Figure 12:**
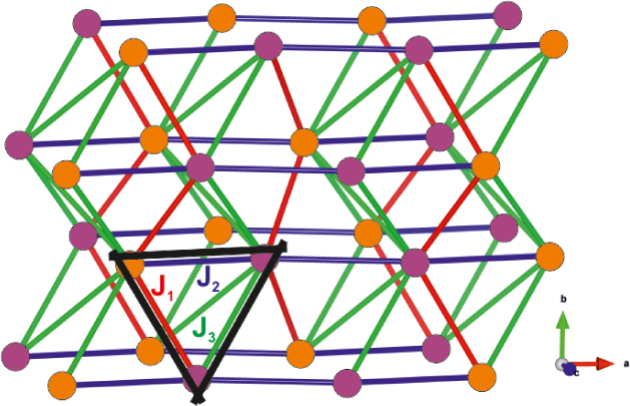
Magnetic model for the Ba_2_FeMS_5_ (M = Sb, Bi) materials. The three shortest Fe–Fe exchange pathways are *J*_1_: red; *J*_2_: blue; *J*_3_: green, and where the distances are in the sequence *J*_1_ < *J*_2_ < *J*_3_. The black triangle highlights an example of a (*J*_1_, *J*_2_, *J*_3_) exchange triangle. The purple and orange dots indicate Fe moments which are collinear and parallel (if they are labeled by the same color) or collinear and antiparallel (if they are labeled by different colors). The Fe moments are directed along the *b*-axis for Ba_2_FeSbS_5_ and along the *a*-axis for Ba_2_FeBiS_5_ as shown in Figures 6 and 10. The model satisfies all the *J*_1_ antiferromagnetic exchange pathways and half of the *J*_2_ and *J*_3_ pathways which are all predicted to be antiferromagnetic.^8^

The arrangements of the Fe^3+^ spins, as summarized in Figures 6 and 10, are dictated by a number of magnetic interactions. In Figures 6 and 10 the magnetic interactions acting over the four shortest Fe-to-Fe distances are shown. It can be seen in these diagrams that the distances over which the interactions act have lengths in the sequence *J*_1_ < *J*_2_ < *J*_3_ < *J*_4_ where the identity of the *J*s is as given in the work of Maier et al.^10^ in their description of Ba_2_FeSbSe_2_. We note here that Koo and Whangbo^8^ consider further exchange pathways in their computational investigation, and their convention is to report the three strongest interactions (*J*_1_, *J*_2_ and *J*_3_ here and in the work of Maier et al.)^10^ as *J*_2_, *J*_4_ and *J*_6_. We adopt the numbering scheme of Maier et al.^10^ because it is related in their paper to a diagram which refers also to the crystallographic axes of the system. Koo and Whangbo^8^ concluded that the strongest magnetic exchange pathways in Ba_2_FeSbS_5_ and Ba_2_FeBiS_5_ are the *J*_1_, *J*_2_ and *J*_3_ defined in Figures 6 and 10 therefore these interactions should dictate the long-range magnetic ordering. The *J*_1_ interactions, which act over the shortest distance, are always antiferromagnetic in the experimental model and they form 1D zigzag chains which run along the *b*-direction (where the structural MS_6_ (M = Sb, Bi) polyhedral chains also run along the *b*-direction). All three of the *J*_1_, *J*_2_ and *J*_3_ exchanges are predicted to be antiferromagnetic so there is magnetic frustration in (*J*_1_, *J*_2_, *J*_3_) exchange triangles as highlighted by the black triangle in Figure 12. In the experimental models, half the *J*_2_ interactions and half of the *J*_3_ interactions are not satisfied as depicted in Figures 6 and 10 and Koo and Whangbo^8^ propose that the lower *T*_N_ for Ba_2_FeSbS_5_ relative to the Weiss temperature arises due to a greater extent of spin frustration in the (*J*_1_, *J*_2_, *J*_3_) triangles in this material compared to Ba_2_FeBiS_5_, originating from their finding that *J*_2_ and *J*_3_ are much closer in magnitude to the dominant interaction, *J*_1_, in Ba_2_FeSbS_5_ than in Ba_2_FeBiS_5_. It seems likely that the additional incommensurate component observed in the magnetic scattering of Ba_2_FeSbS_5_ is a means of easing these highly frustrated (*J*_1_, *J*_2_, *J*_3_) triangles.

The *J*_1_ pathways operate via Fe–S···S–Fe super-superexchange, involving Fe 3*d* orbitals (each occupied by one electron) and S 3*p* orbitals, which result in an exchange that is antiferromagnetic in nature as depicted in Figure S6. The arrangement of the atoms and bonds in this super-superexchange interaction operating along the *J*_1_ pathways, as well as those operating along the *J*_2_ and *J*_3_ pathways, are shown in Figure S7. The *J*_2_ pathways also operate over similar Fe–S···S–Fe super-superexchange pathways, as depicted in Figure S7b. The *J*_3_ pathway can either be viewed as Fe–S···S–Fe super-superexchange or as a Fe–S-M–S-Fe super-superexchange, as illustrated in Figure S7c. Initial findings on the selenide analogues Ba_2_FeMSe_5_ (M = Sb, Bi) presented by J. Wang et al.^9^ give larger Weiss temperatures (see Table 2) and the compounds order at higher temperatures than the sulfides *T*_N_ = 58(1) K for Ba_2_FeSbSe_5_ and *T*_N_ = 79(2) K for Ba_2_FeBiSe_5_. This is presumably due to the stronger super-superexchange interactions along the *J*_1_ Fe–Se···Se–Fe pathways compared to Fe–S···S–Fe pathways. This likely arises because of the greater degree of covalency in the selenide compounds as the Se 4*p* orbitals are more radially extended than the S 3*p* orbitals, resulting in a greater degree of orbital overlap.

The arrangement of antiferromagnetic and ferromagnetic alignment of moments we find in our magnetic model for Ba_2_FeBiS_5_ (Figure 10) is predicted to be the most energetically stable for that compound for all values of the on-site repulsion, *U*, according to the computational studies performed by Koo and Whangbo^8^ (where the values of *U* considered were 1.5, 2.5, 3.0, 4.0, and 5.0 eV). For Ba_2_FeSbS_5_, Koo and Whangbo^8^ predict competition between the model we find experimentally (Figure 6) and a model with an alternative spin arrangement–one where not all *J*_1_ interactions are antiferromagnetic but where every *J*_2_ interaction is antiferromagnetic. The model we find experimentally for Ba_2_FeSbS_5_ in Figure 6 is favored computationally for larger *U* (4.0 and 5.0 eV).^8^ It is plausible that the observation of the incommensurate magnetic peaks, suggestive of a slightly more complex magnetic structure, may be a signature of this competition but single-crystal neutron diffraction would be required to examine this further.

The long-range magnetic ordering which exists in the analogous Ba_2_FeSbSe_5_ selenide has been probed using NPD studies by Maier et al.^10^ They find a magnetic model similar to that shown in Figure 6 for Ba_2_FeSbS_5_, where the Fe^3+^ moments are aligned along the *b*-axis. However, in the case of the selenide, the data presented in reference 10, which have a similar signal-to-noise ratio as ours, show no evidence for any magnetic peaks that cannot be indexed on the commensurate magnetic model. So, as Koo and Whangbo point out,^8^ Ba_2_FeSbS_5_ is unusual.

## Conclusions

The small band gap quaternary sulfides Ba_2_FeSbS_5_ and Ba_2_FeBiS_5_ have been synthesized as black powders on a multigram scale and their structure and magnetic properties, including the long-range magnetic ordering have been investigated experimentally for comparison with computational work^8^ and earlier experimental observations on these and related compounds.^6,9−11^ They contain Fe^3+^ in FeS_4_ tetrahedra and M^3+^ (M = Sb, Bi) in MS_6_ distorted octahedra. The MS_6_ polyhedra are edge-sharing and also share edges with the FeS_4_ polyhedra, which are positioned in an alternating manner along the MS_6_ polyhedral chains. The lone pair of the nontransition metal cation causes the unusual coordination of the sulfide anions around the Sb and Bi centers observed for these compounds and arises due to a second order Jahn–Teller distortion involving ns/np mixing mediated by the anion valence orbitals.^19^

High-resolution NPD data gathered on Ba_2_FeSbS_5_ and Ba_2_FeBiS_5_ reveal that both of these compounds exhibit long-range antiferromagnetic ordering with Néel temperatures of 13(1) K and 35(1) K respectively and it has been found that the magnetic Bragg peaks are located on a *k*-vector (1/2 0 1/2). The arrangement of the Fe^3+^ moments in the magnetic models can be rationalized by considering that *J*_1_ – the strongest antiferromagnetic magnetic interaction (as predicted by computation^8^) and the interaction which acts over the shortest Fe–Fe distance–is always satisfied. This interaction is most likely driven by Fe–S···S–Fe super-superexchange pathways. The Fe^3+^ moment orientations are orthogonal between the two cases, for reasons that are not straightforward to interpret, but reflecting the fact that the Fe^3+^*d*^5^*S* = 5/2 ion has a magnetic moment with only a weak directional preference, as detailed by Whangbo et al.^28^

An unexpected discovery in this work was the appearance of two low-intensity magnetic reflections in the neutron powder diffraction data for Ba_2_FeSbS_5_, which appear at the same temperature as the main magnetic reflections, and suggest an incommensurate component to the ordering which is not found in Ba_2_FeBiS_5_. Single crystal neutron diffraction would be required to obtain a unique solution for the magnetic structure which accounts for these weak reflections in Ba_2_FeSbS_5_, which likely originates from the fact that the magnetic structure is inherently frustrated and that there are competing magnetic ground states suggested by computation.^8^

## Supporting Information Available

Further tables of refinement parameters, fits to magnetometry data, and depictions of magnetic exchange interactions (PDF)
