# Supplementary material for: Contrasting Magnetic Structures in the Quaternary Sulfides Ba2FeMS5 (M = Sb, Bi)
Source: Inorg Chem. 2024 Nov 25;63(49):23267–75. doi: 10.1021/acs.inorgchem.4c03770 (PMC11632765; doi:10.1021/acs.inorgchem.4c03770)
Supplement: Supplementary file 1 — ic4c03770_si_001.pdf [file ic4c03770_si_001.pdf]

Supporting Information

Contrasting magnetic structures in the quaternary sulfides Ba_2FeMS_5 (M = Sb, Bi)

Bradley C. Sheath,[a] Stanislav Savvin,[b,c] and Simon J. Clarke[a,]*

[a]*Department of Chemistry, University of Oxford, Inorganic Chemistry Laboratory, South Parks Road, Oxford OX1 3QR, United Kingdom*

[b]*Institut Laue-Langevin, 71 Avenue des Martyrs CS 20156, 38042 Grenoble Cedex 9, France*

[c]*Instituto de Nanociencia y Materiales de Aragón, CSIC – Universidad de Zaragoza, Facultad de Ciencias C/ Pedro Cerbuna 12, Zaragoza, Spain*

email address: simon.clarke@chem.ox.ac.uk

Table S1a. Refined crystal structure parameters for Ba_2FeSbS_5. Refinements against the D2B (ILL) data (Figure 8) and the I11 (MAC) data (Figure 1) were both performed using isotropic displacement parameters for all atoms.

	Ba_2FeSbS_5 (RMM=612.6, *Z*=4)	
Diffractometer	D2B (ILL)	I11 (Diamond)
Wavelength (Å)	1.594	0.824512
Radiation	Neutron	X-ray
d-space Range (Å)	0.85-10.1	1.2-18.9
Temperature (K)	2	300
Crystal System	Orthorhombic	
Space Group	*Pnma* (62)	
a (Å)	12.0828(3)	12.08609(3)
b (Å)	8.83773(15)	8.834255(18)
c (Å)	8.88054(17)	8.891135(18)
V (Å^3)	948.31(3)	949.321(3)
x[Ba]	0.32260(17)	0.32277(6)
x[Sb]	0.0228(3)	0.02265(9)
x[Fe]	0.09487(12)	0.09434(18)
x[S1]	0.0537(3)	0.0527(3)
x[S2]	0.2108(5)	0.2099(4)
x[S3]	0.2762(5)	0.2737(4)
x[S4]	0.4923(5)	0.4936(4)
y[Ba]	0.0072(3)	0.00774(9)
y[S1]	0.0474(5)	0.0501(3)
z[Ba]	0.3776(3)	0.37767(6)
z[Sb]	0.5175(4)	0.51819(13)
z[Fe]	0.1695(2)	0.1705(3)
z[S1]	0.3212(5)	0.3236(3)
z[S2]	0.6186(7)	0.6157(5)
z[S3]	0.1127(7)	0.1173(5)
z[S4]	0.5387(6)	0.5347(5)
Ba U_{iso} (Å^2)	0.0000(5)	0.0160(3)
Sb U_{iso} (Å^2)	0.0000(7)	0.0134(4)
Fe U_{iso} (Å^2)	0.0000(5)	0.0066(8)
S1 U_{iso} (Å^2)	0.0020(9)	0.0154(11)
S2 U_{iso} (Å^2)	0.0036(12)	0.0118(15)
S3 U_{iso} (Å^2)	0.0034(13)	0.0184(16)
S4 U_{iso} (Å^2)	0.0009(12)	0.0181(14)
χ^2	2.346	1.768
R_p	2.051	6.056
R_{wp}	2.650	7.732

Table S1b. Selected metal-anion bond lengths from the refinements in Table S1a.

Ba_2FeSbS_5	2K neutron	300 K X-ray
Fe–S1 (Å) [×2]	2.295(5)	2.286(3)
Fe–S3 (Å) [×1]	2.248(6)	2.219(5)
Fe–S4 (Å) [×1]	2.226(6)	2.193(5)
Sb–S1a (Å) [×2]	2.527(5)	2.499(3)
Sb–S1b (Å) [×2]	3.133(5)	3.136(3)
Sb–S2 (Å) [×1]	2.443(6)	2.424(4)
Sb–S3 (Å) [×1]	3.196(6)	3.241(4)

Table S2a. Refined crystal structure parameters for Ba_2FeBiS_5. Refinements against the D2B (ILL) data (Figure 14) and the I11 (MAC) data (Figure 2) were both performed using isotropic displacement parameters for all atoms.

	Ba_2FeBiS_5 (RMM=699.8, *Z*=4)	
Diffractometer	D2B (ILL)	I11 (Diamond)
Wavelength (Å)	1.594	0.824512
Radiation	Neutron	X-ray
d-space Range (Å)	0.85-10.1	1.2-18.9
Temperature (K)	2	300
Crystal System	Orthorhombic	
Space Group	*Pnma* (62)	
a (Å)	12.0946(3)	12.09610(3)
b (Å)	8.89802(15)	8.892814(18)
c (Å)	8.81197(17)	8.824371(19)
V (Å^3)	948.32(3)	949.224(3)
x[Ba]	0.32224(18)	0.32224(7)
x[Bi]	0.01707(16)	0.01744(7)
x[Fe]	0.09456(14)	0.0942(3)
x[S1]	0.0547(4)	0.0551(3)
x[S2]	0.2162(6)	0.2159(4)
x[S3]	0.2781(6)	0.2760(4)
x[S4]	0.4943(6)	0.4963(4)
y[Ba]	0.0087(3)	0.00985(10)
y[S1]	0.0449(5)	0.0457(4)
z[Ba]	0.3827(3)	0.38338(9)
z[Bi]	0.5324(3)	0.53310(8)
z[Fe]	0.1706(3)	0.1715(3)
z[S1]	0.3173(6)	0.3199(4)
z[S2]	0.6260(8)	0.6245(6)
z[S3]	0.1148(7)	0.1177(6)
z[S4]	0.5384(8)	0.5345(5)
Ba U_{iso} (Å^2)	0.0001(6)	0.0042(4)
Bi U_{iso} (Å^2)	0.0005(5)	0.0044(4)
Fe U_{iso} (Å^2)	0.0007(5)	0.0000(9)
S1 U_{iso} (Å^2)	0.0007(9)	0.0028(12)
S2 U_{iso} (Å^2)	0.0012(16)	0.0027(17)
S3 U_{iso} (Å^2)	0.0019(16)	0.0017(16)
S4 U_{iso} (Å^2)	0.0049(13)	0.0050(14)
χ^2	2.817	1.376
R_p	2.246	6.503
R_{wp}	2.936	8.401

Table S2b. Selected metal-anion bond lengths from the refinements in Table S2a.

Ba_2FeBiS_5	2K neutron	300 K X-ray
Fe–S1 (Å) [×2]	2.288(5)	2.289(4)
Fe–S3 (Å) [×1]	2.274(7)	2.250(6)
Fe–S4 (Å) [×1]	2.205(7)	2.170(6)
Bi–S1[a] (Å) [×2]	2.670(5)	2.655(4)
Bi–S1[b] (Å) [×2]	3.065(5)	3.061(4)
Bi–S2 (Å) [×1]	2.546(7)	2.532(5)
Bi–S3 (Å) [×1]	3.168(7)	3.209(5)

Table S3. Summary of the magnetic structures of Ba_2FeSbS_5 and Ba_2FeBiS_5 which were treated as separate magnetic-only phases with lattice parameters and atomic positions tied to the nuclear structure in the refinements against 1.9 K D1B data (Figures 7 and 11). Shubnikov groups are given in the BNS notation. Fe1 and Fe2 are on two 4*e* sites of the magnetic space groups, but correspond to a single 4*c* site in the nuclear *Pnma* cell.

Compound	Ba_2FeSbS_5	Ba_2FeBiS_5
Space group	P_a2_1/m (11.55)	P_c2_1/c (14.82)
a (Å)	17.696(1)	14.917(1)
b (Å)	8.8066(7)	8.8692(5)
c (Å)	14.942(1)	17.569(1)
β (°)	126.31(2)	126.08(2)
V (Å^3)	1876.4(1)	1878.5(1)
Fe1(*x*)	0.618(1)	0.594(1)
Fe1(*y*)	0.25	0.25
Fe1(*z*)	0.906(1)	0.211(1)
Fe1($\mathbf{M}_x$)	0	4.68(3)
Fe1($\mathbf{M}_y$)	3.56(2)	0
Fe1($\mathbf{M}_z$)	0	2.76(2)
Fe2(*x*)	0.288(1)	0.094(1)
Fe2(*y*)	0.25	0.25
Fe2(*z*)	0.406(1)	0.383(1)
Fe2($\mathbf{M}_x$)	0	–4.68(3)
Fe2($\mathbf{M}_y$)	–3.56(2)	0
Fe2($\mathbf{M}_z$)	0	–2.76(2)
|**M**| (Fe1 & Fe2)	3.56(2)	3.78(3)

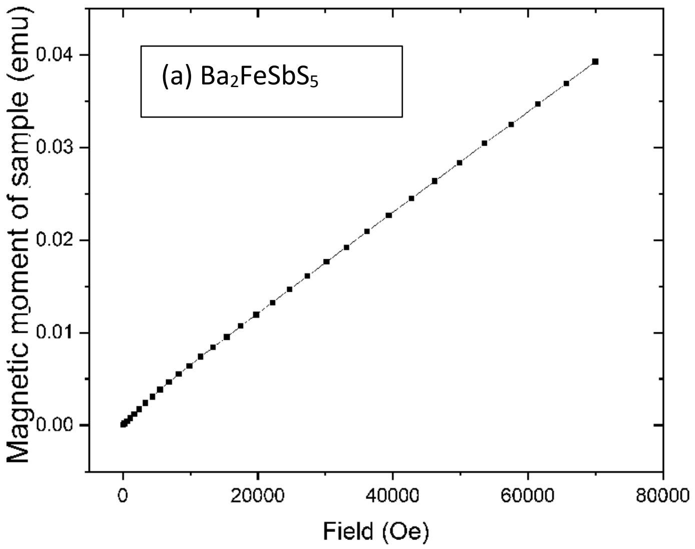

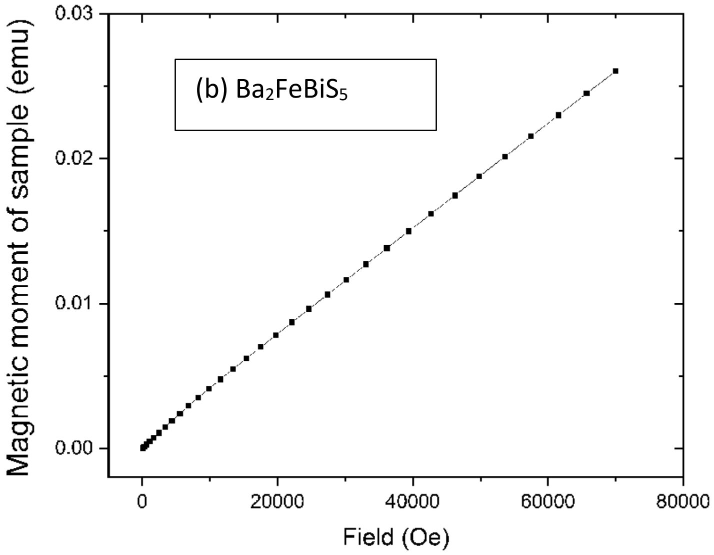

Figure S1. Magnetisation isotherms (raw data as measured in the magnetometer) at 300 K for (a) Ba_2FeSbS_5 (21.7 mg sample) and (b) Ba_2FeBiS_5 (20.7 mg sample) showing very slight curvature at low fields consistent with a small elemental Fe or other ferromagnetic impurities. Magnetic susceptibilities were determined from the gradients in the region between 3T and 5T where the gradients are linear above the magnetic ordering transitions.

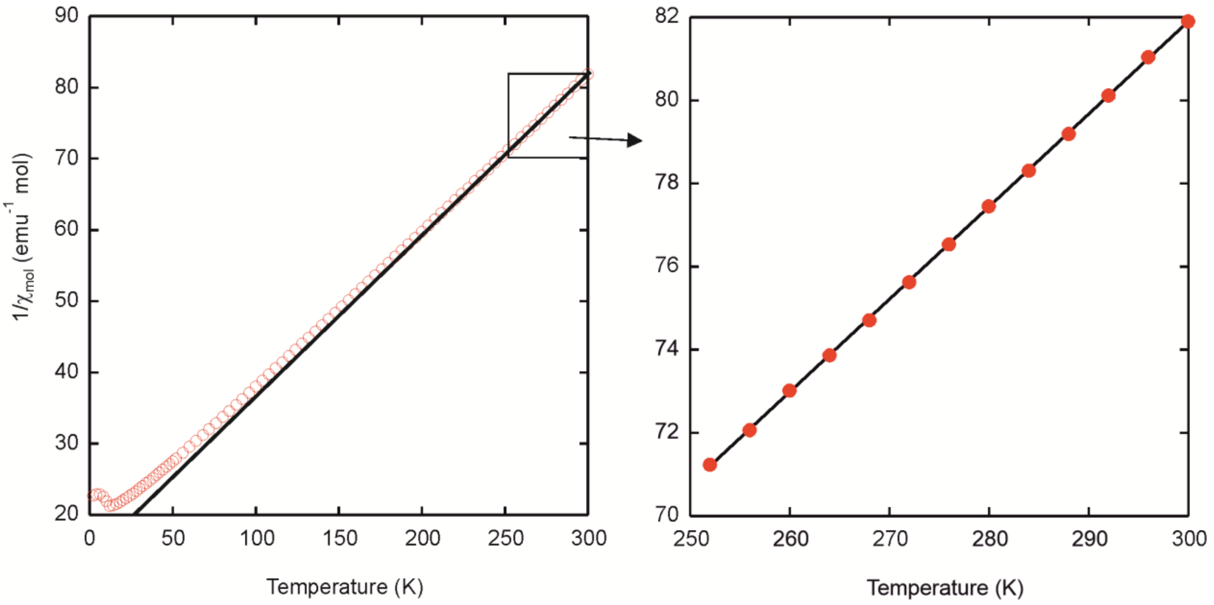

Figure S2 Curie-Weiss fit for the magnetometry data for Ba_2FeSbS_5. The fit (right) range was 250 K to 300 K because there were deviations from linearity below that temperature (left) in line with the Weiss temperature which was determined to be –67.5(5) K. The effective magnetic moment, μ_{eff}, per Fe^{3+} ion was 5.990(3) μ_B.

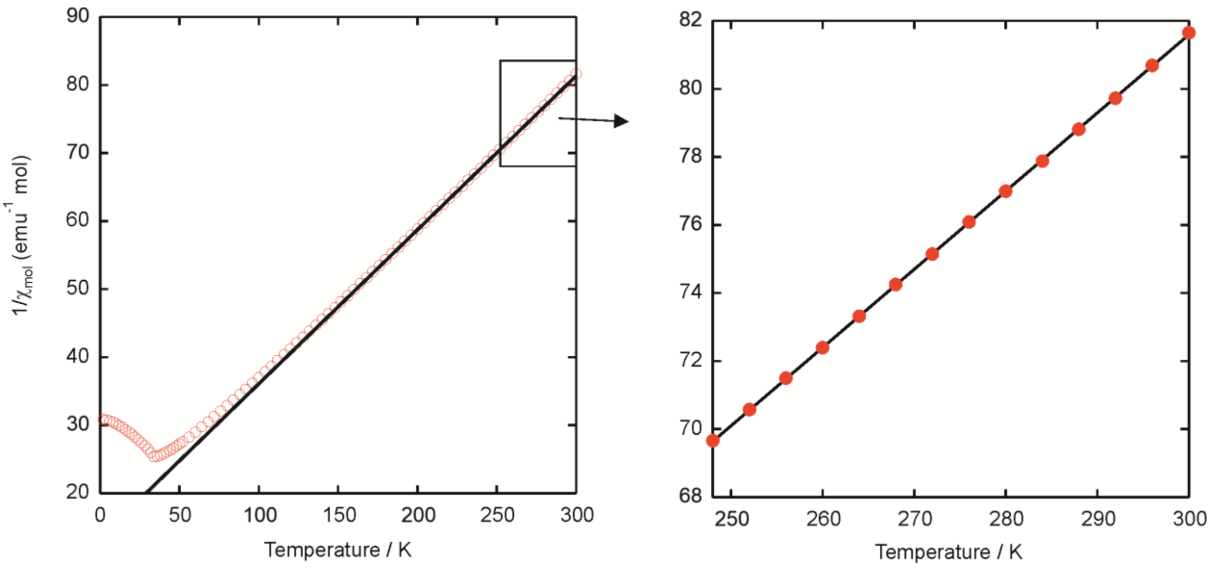

Figure S3 Curie-Weiss fit for the magnetometry data for Ba_2FeBiS_5. The fit range was 250 K to 300 K because there were deviations below that temperature in line with the Weiss temperature which was determined to be –55.3(5) K. The effective magnetic moment, μ_{eff}, per Fe^{3+} ion was 5.902(3) μ_B.

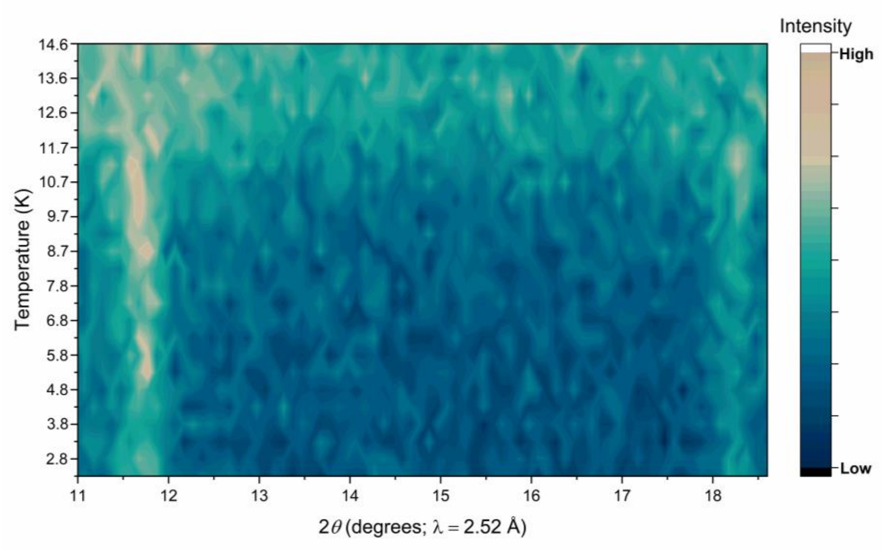

Figure S4. Plot of the NPD data collected for Ba_2FeSbS_5 from 2 K to 15 K on D1B at the ILL focussing on the 2θ range which shows the evolution of the two small, incommensurately modulated magnetic Bragg peaks (occurring at approximately 2θ = 11.7° and 2θ = 18.3°). They appear at the same temperature as the other magnetic Bragg peaks where the magnetic scattering crystallises into Bragg peaks (hence the drop in the background scattering below 12 K in the figure above).

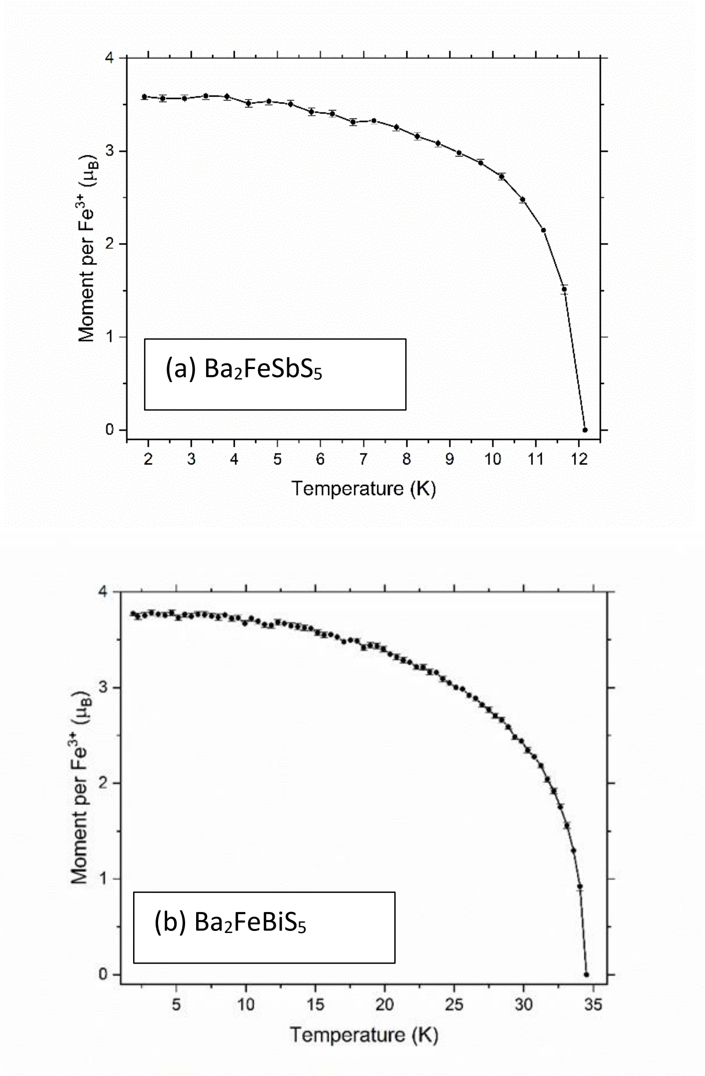

Figure S5. Plot depicting the thermal evolution of the long-range ordered Fe^{3+} magnetic moment in and (b) Ba_2FeBiS_5 refined from data collected on D1B at the ILL.

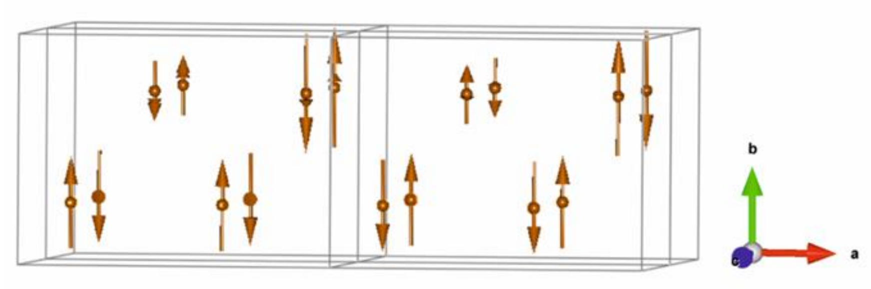

Figure S6. Schematic of a magnetic model for Ba_2FeSbS_5 which includes an incommensurately modulated spin-density wave with wavevector k = 0.186 ½ ½ superimposed on the commensurate antiferromagnetic ordering k = ½ 0 ½ . Nuclear unit cells are depicted. In view of the small number of additional reflections found, this is not the only possible solution, and single crystal neutron diffraction data would be required to solve this unambiguously.

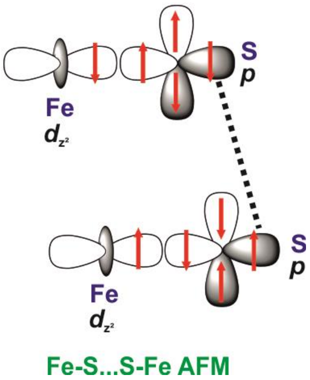

Figure S7. Schematic of the Fe 3*d* and S 3*p* orbitals involved in the Fe-S…S-Fe super-superexchange interactions. These interactions acting along the shortest J_1 pathway are predicted[1] (reference 8 in main article) to be the driving force for the antiferromagnetic ordering observed in the Ba_2FeMS_5 (M = Sb, Bi) materials. The diagram depicts these orbitals in their relative arrangements as they exist in the structures.

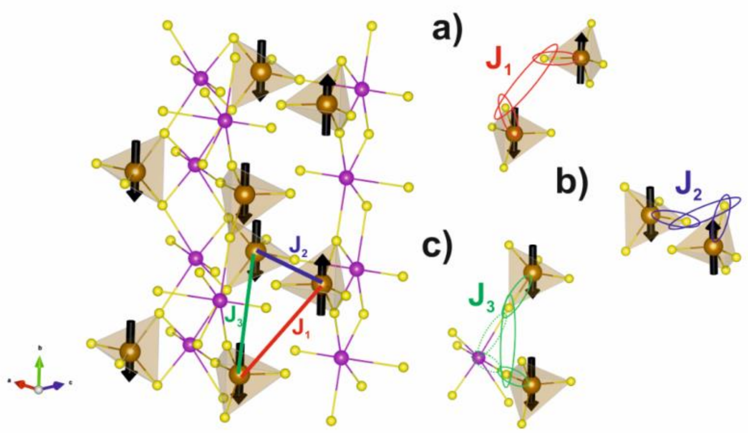

Figure S8. A portion of the magnetic model for Ba_2FeSbS_5, showing the connecting Sb (purple) and S (yellow) atoms between the Fe (brown) centres. The super-superexchange interactions, as discussed by Koo and Whangbo,[1] (reference 8 in main article) highlighted are **a)** the Fe-S…S-Fe J_1 pathway (solid red ovals) **b)** the Fe-S…S-Fe J_2 pathway (blue solid ovals) and **c)** the Fe-S…S-Fe J_3 pathway (solid green ovals) and the Fe-S-Sb-S-Fe J_3 pathway (via dashed green ovals). All the interactions are predicted to favour antiferromagnetism, but cannot all be satisfied. The labels of the *J* pathways are as used by Maier *et al.*[2] (ref 10 in the main article) as discussed in the main text.

References

[1] Koo, H. J.; Whangbo, M. H. Density Functional Investigation of Why Ba_2BiFeS_5 and Ba_2SbFeS_5 Differ in Their Magnetic Properties. *J. Magn. Magn. Mater.* **2014**, *360*, 152–156. https://doi.org/10.1016/j.jmmm.2014.02.024.

[2] Maier, S.; Gaultois, M. W.; Matsubara, N.; Surta, W.; Damay, F.; Hebert, S.; Hardy, V.; Berthebaud, D.; Gascoin, F. Sb-5s Lone Pair Dynamics and Collinear Magnetic Ordering in $Ba_2FeSbSe_5$. *Phys. Rev. B* **2021**, *103*, 54115. https://doi.org/10.1103/PhysRevB.103.054115.
